# Bayesian Phylogeography of Crimean-Congo Hemorrhagic Fever Virus in Europe

**DOI:** 10.1371/journal.pone.0079663

**Published:** 2013-11-04

**Authors:** Gianguglielmo Zehender, Erika Ebranati, Renata Shkjezi, Anna Papa, Camilla Luzzago, Elena Gabanelli, Alessandra Lo Presti, Alessia Lai, Giovanni Rezza, Massimo Galli, Silvia Bino, Massimo Ciccozzi

**Affiliations:** 1 Department of Clinical Sciences “Luigi Sacco”, Section of Infectious Diseases, University of Milan, Milano, Italy; 2 Faculty of Medicine, Catholic University “Our Lady of Good Council”, Tirana, Albania; 3 Department of Microbiology, Medical School, Aristotle University of Thessaloniki, Thessaloniki, Greece; 4 Department of Veterinary Science and Public Health, University of Milan, Milano, Italy; 5 Epidemiology Unit, Department of Infectious Parasitic and Immunomediate Diseases, Istituto Superiore di Sanità, Roma, Italy; 6 Control of Infectious Diseases Department, National Institute of Health, Tirana, Albania; Instituto de Higiene e Medicina Tropical, Portugal

## Abstract

Crimean-Congo hemorrhagic fever (CCHF) is a zoonosis mainly transmitted by ticks that causes severe hemorrhagic fever and has a mortality rate of 5-60%. The first outbreak of CCHF occurred in the Crimean peninsula in 1944-45 and it has recently emerged in the Balkans and eastern Mediterranean. In order to reconstruct the origin and pathway of the worldwide dispersion of the virus at global and regional (eastern European) level, we investigated the phylogeography of the infection by analysing 121 publicly available CCHFV S gene sequences including two recently characterised Albanian isolates. The spatial and temporal phylogeny was reconstructed using a Bayesian Markov chain Monte Carlo approach, which estimated a mean evolutionary rate of 2.96 x 10^-4^ (95%HPD=1.6 and 4.7 x 10^-4^) substitutions/site/year for the analysed fragment. All of the isolates segregated into seven highly significant clades that correspond to the known geographical clades: in particular the two new isolates from northern Albania clustered significantly within the Europe 1 clade. Our phylogeographical reconstruction suggests that the global CCHFV clades originated about one thousand years ago from a common ancestor probably located in Africa. The virus then spread to Asia in the XV century and entered Europe on at least two occasions: the first in the early 1800s, when a still circulating but less or non-pathogenic virus emerged in Greece and Turkey, and the second in the early 1900s, when a pathogenic CCHFV strain began to spread in eastern Europe. The most probable location for the origin of this European clade 1 was Russia, but Turkey played a central role in spreading the virus throughout Europe. Given the close proximity of the infected areas, our data suggest that the movement of wild and domestic ungulates from endemic areas was probably the main cause of the dissemination of the virus in eastern Europe.

## Introduction

Crimean-Congo hemorrhagic fever virus (CCHFV) belongs to the family *Bunyaviridae*, genus *Nairovirus*. It is an enveloped virus with a negative-sense single stranded RNA genome consisting of one small (S), one medium (M) and one large segment (L) that respectively encode the viral nucleocapsid (N), the membrane glycoprotein precursor (GPC), and RNA-dependent RNA polymerase (L) proteins [1,2].

Crimean-Congo hemorrhagic fever is a mainly tick-borne zoonosis that causes sporadic cases and severe outbreaks of acute human disease with a mortality rate of 5-60% depending on the country [3,4]. The genus *Hyalomma*, particularly the *H. marginatum* of *Ixodes* ticks, is the principal vector of CCHFV, and its geographical distribution correlates with the occurrence of CCHF [4]. In addition to *Hyalomma* spp., the transmission of CCHFV has been associated with *Rhipicephalus* (Europe, South Russia), *Boophilus* (Bulgaria, Russia, Pakistan), *Dermacentor* (Europe), *Ixodes* spp. (Crimea, Moldavia, Bulgaria, Hungary) [5], *Argas persicus* (Uzbekistan) and *Ornithodoros lahorensis* (Iran) [6,7]. Various wild and domestic mammals act as hosts for feeding ticks that can be infected with the virus; the animal infection is generally asymptomatic.

The disease’s seasonal pattern (between spring and early autumn) reflects the high degree of tick activity during this period [8]. Other modes of transmission are direct contact with infected animal blood, viremic CCHF patients and nosocomial infection [9,10].

CCHFV is endemic in parts of Eurasia and Africa, and continues to spread. A number of publications have demonstrated the presence of the virus in about 18 countries in Africa, 11 in South Asia and the Middle East, and eight in eastern Europe [6,8,11]. 

The migration or transportation of tick-infested birds [4], movements of livestock [12], the environment, tick density, the number of host vertebrate animals [13], and climatic and agricultural changes have all played major roles in taking CCHFV into new areas [8]. The first outbreak of severe hemorrhagic fever associated with CCHFV in Europe (and the world) occurred in the Crimean peninsula (on the Ukrainian coast of the Black Sea) in 1944-45, when hundreds of Soviet soldiers were infected [14]. Other large outbreaks have subsequently been described in Bulgaria (since 1953) and in the regions of Astrakhan and Rostov in south-eastern European Russia [8]. Recent studies in Greece have shown that the seroprevalence of already circulating and new-entry forms has increased from 1.1% to 3.14% over the last 20 years [15]; most of the detected antibodies were against the AP92 strain (isolated in 1970s), which is genetically different from all of the other strains and seems to be less or non-pathogenic to humans. Since 2002, Turkey has reported an exponentially growing number of human cases, whereas seroprevalence has decreased in Bulgaria since the 1974 introduction of an immunisation programme targeting military personnel and healthcare workers [8]. Kosovo experienced a reactivation of natural foci and the re-emergence of CCHF during the 1990s because of dramatic changes in social, political, environmental and economic factors that may together have led to more suitable ecological conditions. There is a seroprevalence of about 24% in the general population living in endemic areas in Kosovo [8,16]. In 2001, there was an outbreak of eight confirmed CCHF cases in Albania at the same time as an epidemic in Kosovo. During 2002-2006, another 24 patients were found to have confirmed CCHF, many of whom lived in Kukës Prefecture, in north-eastern Albania, which shares a border with Kosova. Another outbreak in the same prefecture was reported in 2010, and a few cases were also detected in the south-eastern part of the country in Kolonja near the border with Greece. Recent epidemiological surveillance has found an overall national seroprevalence in Albania of 2.1%, with significant differences between districts ranging from 0.8 to 12% (Silvia Bino, personal communication, 2013).

Analyses of the viral S and L-RNA segments have led to the identification of seven major genetic clades that segregate on a geographical basis [1,17-22]: Africa 1 (also classified as genotype 7), Africa 2 (genotype 5), Africa 3 (genotype 3), Asia 1 (genotype 1), Asia 2 (genotype 2), Europe 1 (genotype 4) and Europe 2 (genotype 6). Analysis of the M segment revealed only six clades, with all of the Asian isolates in a single monophyletic group [23]. 

We phylogenetically analysed publicly available S region sequences of CCHFV isolated in different geographical areas at different times (including two recently characterized isolates obtained in Albania) in order to reconstruct the most probable places of origin and pathways of dispersion of the CCHFV clades, particularly concentrating on the strain causing the recent outbreaks in eastern Europe.

## Materials and Methods

### Sequence data sets

The analysis included a total of 121 S gene sequences of CCHFV isolated in various countries of the world and retrieved from public databases (Genbank at http://www.ncbi.nlm.nih.gov/genbank/). The sampling dates ranged from 1958 to 2010, and the sampling locations were Albania (n=2), Turkey (n=37), Russia (n=5), China (n=15), Central Asia (n=4), Iran (n=14), Iraq (n=1), Oman (n=1), Africa (n=31), Pakistan (n=3), Afghanistan (n=1), Kosovo (n=3), Bulgaria (n=2) and Greece (n=2). Accession numbers and main characteristics of the isolates are reported in Table S1. The two Albanian sequences isolated during the recent outbreaks, have been characterized and submitted to GenBank (assigned accession numbers: KC846093 for AL6@03 and KC846094 for AL5@04). 

All of the sequences were aligned using ClustalX software [24] and then manually edited using Bioedit software v. 7.0 (freely available at http://www.mbio.ncsu.edu/bioedit/bioedit.html).

### Likelihood mapping

In order to obtain an overall impression of the phylogenetic signal present in the analysed CCHFV S sequences, we made a likelihood-mapping analysis of 10,000 random quartets generated using TreePuzzle [25]. A likelihood map consists of an equilateral triangle: each dot within the triangle represents the likelihood of the three possible unrooted trees for a set of four sequences (quartets) randomly selected from the dataset. The dots close to the corners and at the sides respectively represent tree-like (fully resolved phylogenies in which one tree is clearly better than the others) and network-like phylogenetic signals (three regions in which it is not possible to decide between two topologies); the central area of the map represents a star-like signal (the region in which the star tree is the optimal tree). 

### Phylogenetic reconstruction

The evolutionary model that best fitted the data was selected using an information criterion implemented in JmodelTest [26], which is freely available at http://darwin.uvigo.es/software/jmodeltest.html. 

The phylogenesis of the S gene sequences was initially analysed using a maximum likelihood approach with a new hill-climbing algorithm implemented in the Phyml server v.3.0 (http://www.atgc-montpellier.fr/phyml/) [27]. The reliability of the observed clades was established on the basis of an internal node bootstrap support value of more than 0.90 (after 200 replicates). 

The evolutionary rates were estimated using a Bayesian Markov Chain Monte Carlo (MCMC) method implemented in BEAST 1.7.4 [28] with a strict and relaxed molecular clock and an uncorrelated log normal rate distribution model (assuming the GTR+G+I model of nucleotide substitution). As coalescent priors, we compared three parametric demographic models of population growth (constant size, exponential growth, and logistic growth) and a a piecewise-constant Bayesian skyline plot (BSP) [29].

The chains were conducted until reaching convergence as described below, and sampled every 10,000 steps. Convergence was assessed on the basis of the effective sampling size (ESS=>200) after a 10% burn-in using Tracer software v. 1.5 (http://tree.bio.ed.ac.uk/software/tracer/). Uncertainty in the estimates was indicated by the 95% highest posterior density (95% HPD) intervals and the best fitting models were selected using a Bayes factor (BF) with marginal likelihoods implemented in BEAST. In accordance with [30], the strength of the evidence against H0 was assessed as 2lnBF <2 no evidence; 2–6 weak evidence; 6–10 strong evidence, and >10 very strong evidence. A negative 2lnBF indicates evidence in favour of H0. Only values of >6 were considered significant. The trees were summarised in a maximum clade credibility (MCC) tree (the tree with the largest product of posterior clade probabilities) after a 10% burn-in using the Tree Annotator program included in the BEAST package. The time of the most recent common ancestor (tMRCA) estimates were expressed as mean and 95% HPD years before the most recent sampling dates (corresponding to 2010 in this study).

### Bayesian phylogeography

The geographical analysis was made using the continuous time Markov chain (CTMC) process over discrete sampling locations implemented in BEAST, and the Bayesian Stochastic Search Variable Selection (BSSVS) model, which allows diffusion rates to be zero with a positive prior probability. Comparison of the posterior to prior odds that the individual rates are non-zero provides a formal BF for testing the signiﬁcance of the linkage between locations [31]. Rates with a BF of > 3 were considered well supported and formed the migration pathway.

The MCC tree was selected from the posterior tree distribution after a 10% burn-in using the TreeAnnotator program, version 1.7.4. The ﬁnal tree was visualised using FigTree version 1.4 (available at http://tree.bio.ed.ac.uk/software). The significant migration rates were analysed and visualised using SPREAD [32], which is available at http://www.kuleuven.be/aidslab/phylogeography/SPREAD.html. The 121 isolates were assigned to a total of 11 distinct geographic groups corresponding to: Africa, Albania, central Asia, Bulgaria, China, Greece, Kosovo, the Middle East (grouping the 16 isolates from Oman, Iran and Iraq), Pakistan (including 4 sequences from Pakistan and Afghanistan), Russia and Turkey. 

In order to provide a spatial projection, the migration routes indicated by the tree were visualised using Google Earth (http://earth.google.com).

In order to investigate a possible bias due to the uneven sample size at each location we performed a sensitivity test involving the randomisation of the tip-location throughout the MCMC procedure (described in more detail in the Figure S1).

The hypothesis of restricted gene flow among distinct CCHFV populations in eastern Europe was investigated in more detail by analysing the European clade using a modified version of the Slatkin and Maddison test [33], which counts migrations to/from different viral subpopulations, using the MacClade version 4 program (Sinauer Associates, Sunderland, MA). A one-character data matrix is obtained from the original data set by assigning a one-letter code to each taxon in the tree indicating its country (or geographic region) of origin, and then the putative origin of each ancestral sequence (i.e. internal node) in the tree is inferred by finding the most parsimonious reconstruction (MPR) of the ancestral character. The final tree length (i.e. the number of observed migrations in the genealogy) can easily be computed and compared with the tree-length distribution of 10,000 trees obtained by means of random joining-splitting. Observed genealogies that are significantly shorter than the random trees indicate the presence of subdivided populations with restricted gene flow. Specific migrations from/to different countries (character states) were traced using the state changes and stasis tool (MacClade software), which counts the number of changes in a tree for each pair-wise character state. In the presence of multiple MPRs (as in our data sets), the algorithm calculates the average migration count over all possible MPRs for each pair. The resulting pair-wise migration matrix was then normalised, and underwent a randomisation test using 10,000 matrices obtained from 10,000 random trees (by means of the random joining-splitting of the original tree) in order to assess the statistical significance of the observed migration counts. The S gene sequences of the CCHF sequences were assigned to six distinct European countries: Albania, Bulgaria, Greece, Kosovo, Russia and Turkey.

## Results

### Likelihood mapping

The phylogenetic noise of the data set was investigated by means of likelihood mapping. The evaluation of 10,000 randomly chosen quartets showed that only 4.3% fell in the central area of the likelihood map, and 91.8% were at the corners of the triangle, which suggested that the alignment contained sufficient phylogenetic information (Figure S2). 

### Phylogenetic analysis

Maximum likelihood analysis of the S segments showed that the isolates segregated into significant clades (Figure S3) corresponding to the seven known clades: Asia 1 Asia 2, Africa 1-3, and Europe 1 and 2. The clades were significantly supported by bootstrap values of ≥90%. As previously described, the Africa 1 clade included mainly (but not exclusively) isolates from western-Africa (Senegal and Mauritania); the Africa 2 clade isolates from central Africa (Uganda, Democratic Republic of the Congo); and the Africa 3 clade isolates from South Africa. The Asia 1 clade included isolates from the Middle East (Oman, Iraq, Pakistan, Afghanistan and Iran), and the Asia 2 clade isolates from the Far East and central Asia (China, Tajikistan, Uzbekistan). The Europe 1 clade included the majority of the eastern European isolates (Russia, Turkey and a single Greek strain), and contained a monophyletic group consisting of the three sequences from Kosovo and the two new Albanian isolates, whereas the Europe 2 clade isolates encompassed the prototype Greek strain AP92 and five Turkish isolates. 

#### Evolutionary rate estimation and dated tree reconstruction

Comparison of the coalescent models using the BF test showed that BSP (2lnBF constant *vs* BSP= 51.9) under the relaxed clock (2lnBF strict *vs* relaxed clock = 23.5) was the model that best fitted the data. A mean evolutionary rate of 2.96 x 10^-4^ substitutions/site/year (credibility interval 1.6-4.7 x 10^-4^ substitutions/site/year) was estimated under these conditions.

**Figure 1:**
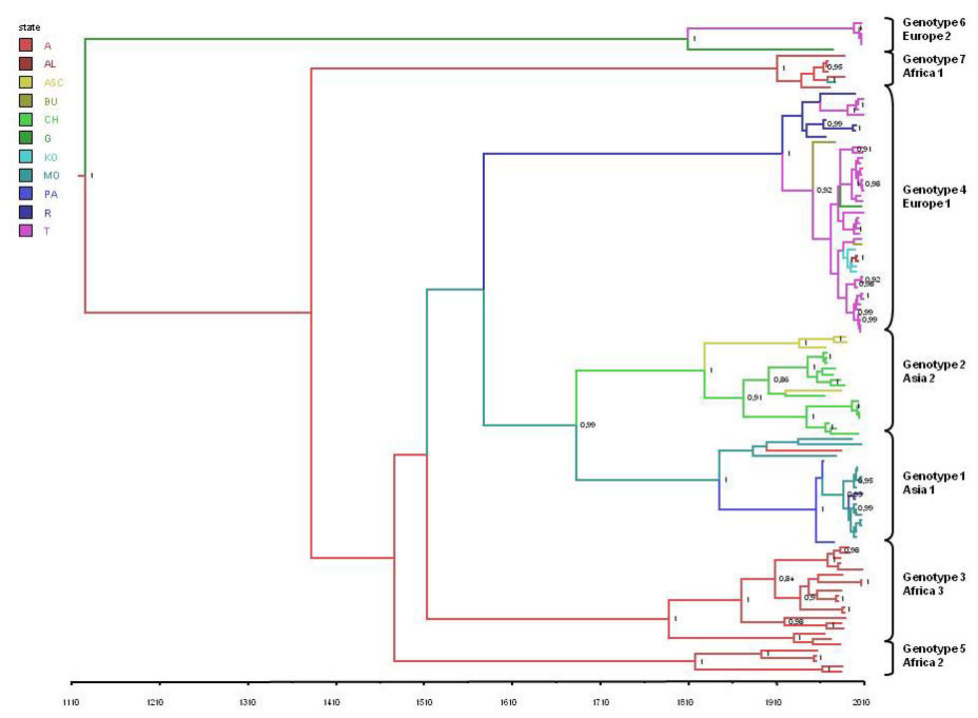
The maximum clade credibility (MCC) tree of CCHFV S gene sequences. The branches are coloured on the basis of the most probable location of the descendent nodes (A=Africa, AL=Albania, ASC=Central Asia, BU=Bulgaria, CH=China, G=Greece, KO=Kosovo, MO=Middle East, PA=Pakistan, T=Turkey). The numbers on the internal nodes indicate significant posterior probabilities (pp>0.8), and the scale at the bottom of the tree represents the number of years before the last sampling time (2010). The main geographical clades (genotypes) have been highlighted.

Figure 1 shows the maximum clade credibility (MCC) tree summarising all of the trees obtained during the MCMC search. All of the seven main clades corresponding to the viral genotypes were confirmed in the Bayesian tree, and supported by posterior probabilities of 1. There were also some highly significant subclades: two main groups in the Europe 1 clade separated the Russian isolates from most of the Turkish and all of the Balkan sequences (the last indicated by blue branches in Figure 1); and subclades in the Asia 2 clade separated the Chinese from the central Asian isolates. Asia 1 and 2 shared a common internal node with a high level of significance in the Bayesian tree (pp=0.99), but not in the ML tree (bootstrap value 0.59). 

Using the above evolutionary rate estimates, we calculated the mean tMRCA for the root and each internal node of the tree (Table 1). The mean tMRCA estimate for the tree root was 884.6 years ago (95%HPD 345-1522 years ago), thus suggesting that CCHFV has a remote origin. The Asian clades originated in the first decades of the 1800s, and a common ancestor of the two clades existed a mean 327 years ago (1683). The tMRCA estimates for the African clades were between 100 and 222 years ago, which suggests that these genotypes radiated between the late 1700s and the early 1900s. Finally, the Europe 1 clade had a mean tMRCA of 93 years ago, thus indicating its relatively recent emergence (mean estimate 1917), and the Europe 2 clade had a mean tMRCA of 201 years ago, corresponding to the first decade of the 1800s. 

**Table 1:** Time of the most recent common ancestor (tMRCA) estimates.

**CLADE**	**Subclade**	**tMRCA^1^**	**CI tMRCA L** ^2^	**CI tMRCA U^3^**	**Locality**	**State pp^4^**
ROOT		884,6	345	1522	AFRICA	0,37
AFRICA 1		164,8	94	302	AFRICA	0,98
AFRICA 2		100	51	166	AFRICA	0,99
AFRICA 3		222,5	109	352	AFRICA	0,98
ASIA 1		164,8	79	261	MIDDLE EAST	0,57
	PAKISTAN	55,5	45	72	PAKISTAN	0,9
	MIDDLE EAST	127	56	204	MIDDLE EAST	0,68
ASIA 2		181,5	97	272	CHINA	0,55
	CHINA	137,29	81	205	CHINA	0,85
	CENTRAL ASIA	74,4	46	109	CENTRAL ASIA	0,93
EUROPE 1		93	57	138	RUSSIA	0,53
	TURKEY	58,5	36	86	TURKEY	0,5
	RUSSIA	71,3	49	99	RUSSIA	0,82
EUROPE 2		201	66	373	GREECE	0,35
ASIA 1+2		326,9	168	514	MIDDLE EAST	0,36

^1^tMRCA: Time of the most recent common ancestor

^2^CI tMRCA L: Lower credibility interval

^3^CI tMRCA U: Upper credibility interval

^4^pp: posterior probability

Time of the most recent common ancestor (tMRCA) estimates and credibility intervals (95%HPD) of the main clades observed in the MCC tree, with the corresponding years, most probable locations, and state posterior probabilities (pp).

### Phylogeographical analysis

As shown in Figure 1, the highest posterior probability for the location of the tree root was Africa (pp=0.4 *vs* pp= 0.19 for the Middle East, the second most probable location). 

The most probable locations for the different clades are shown in Table 1. In particular, the MRCA shared by the two Asian clades was located in the Middle East (pp=0.36 *vs* 0.25 for Pakistan), as was that of the clade Asia 1 (pp=0.57 vs. pp=0.34 for Pakistan). On the contrary, the MRCA of the second Asian clade (Asia 2) was located in China (pp=0.55 *vs* pp=0.38 for central Asia). Interestingly, the MRCA of the Europe 1 clade was more probably located in Russia (pp=0.53 *vs* pp=0.22 for Turkey). The majority of the Turkish isolates segregated into a specific subclade that also included the isolates from Kosovo and Albania (pp=0.55). A Bulgarian isolate was at the outgroup of the Turkish MRCA. Finally, the Europe 2 clade most probably originated in Greece (pp=0.35 *vs* 0.23 for Turkey). 

**Figure 2:**
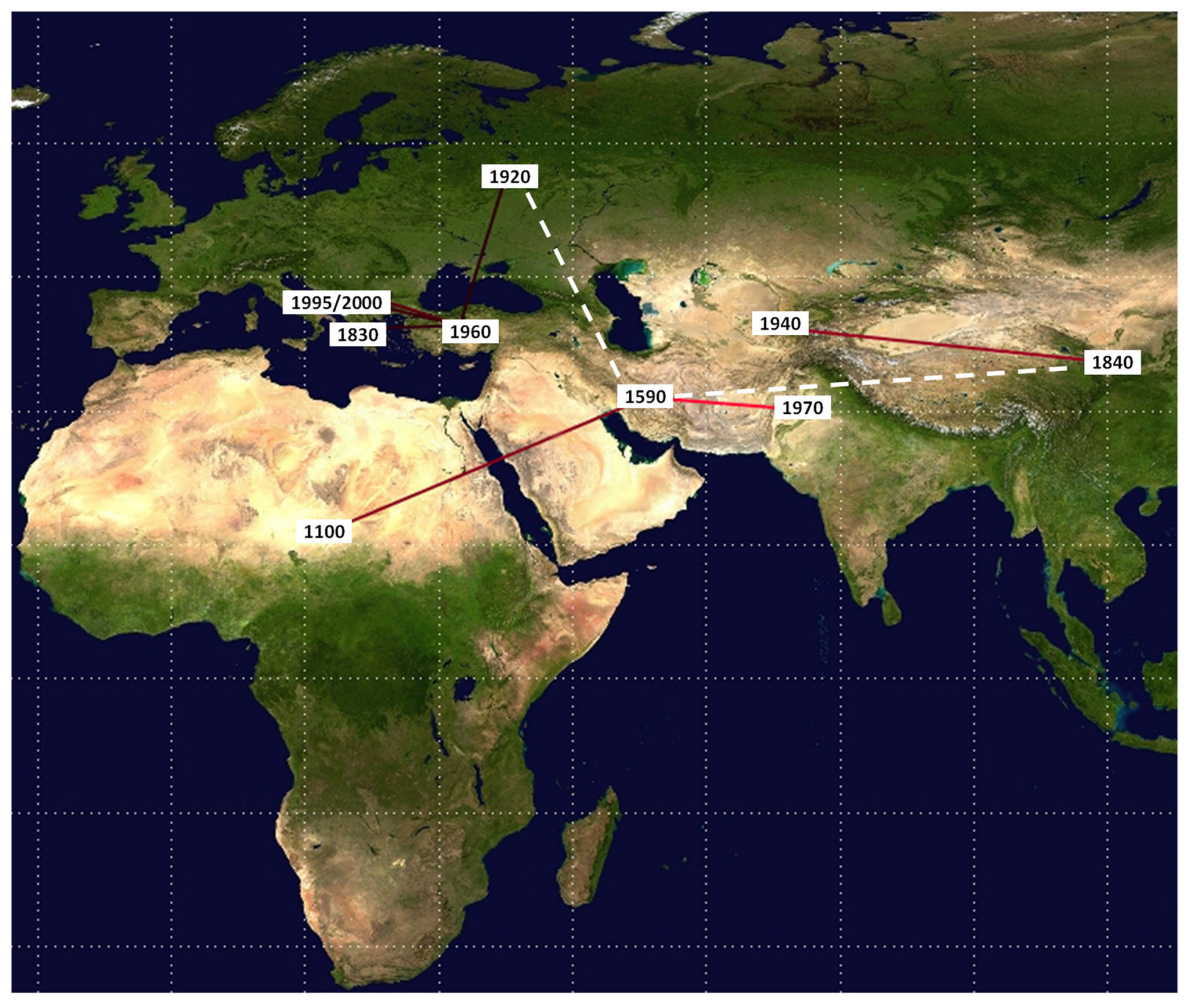
Significant non-zero CCHFV migration rates worldwide. Rates supported by a BF of >3 are highlighted: the relative strength of the support is indicated by the colour of the lines (from dark red = weak to light red = strong). Dotted lines indicate non-significant linkages. The map was reconstructed using SPREAD (see Methods). The numbers indicate the mean estimated year in which the virus entered the area.

Bayesian phylogeographical analysis found a mean of 10.5 non-zero rates (95%HPD=10-12), and the rates with a BF of >3 were those between Africa and the Middle East (BF=48.9), the Middle East and Pakistan (BF=2518), China and Central Asia (BF=91), Russia and Turkey (BF=4.7), Turkey and Kosovo (BF=6.0), Kosovo and Albania (BF=12.1), and Bulgaria and Turkey (BF=16.4). The migration routes are shown in Figure 2. 

**Figure 3:**
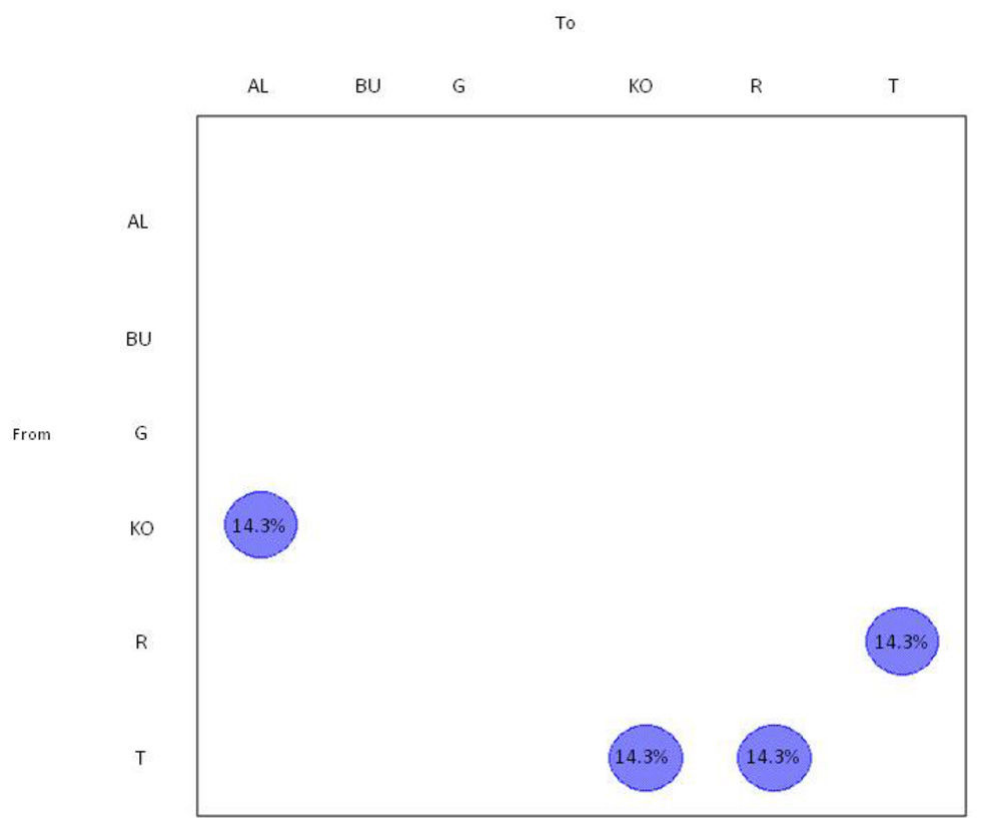
Phylogeographical mapping of CCHF S gene sequences . The bubblegrams show the frequency of gene flows (migrations) to/from ten European countries (same code as that used in Figure 1) . The surface of each circle is proportional to the percentage of observed migrations in the ML genealogy. The migrations were inferred using a modified version of the Slatkin and Maddison algorithm.

The gene flow (migration) of CCHFV between different European areas was also investigated by analysing the European clade alone using a modified version of the Slatkin and Maddison method. The null hypothesis of panmixia (i.e. no population subdivision or the complete intermixing of sequences from different geographical areas) was tested using a bubblegram (Figure 3), and was rejected for Kosovo, Russia, Turkey and Bulgaria by the randomisation test (p = 0.0001).

Almost 14.3% of gene flow was from Kosovo to Albania and from Turkey to Kosovo. Interestingly, there was a bidirectional gene flow between Turkey and Russia. 

## Discussion

Various authors have studied the genetic heterogeneity of CCHFV worldwide and found that the existing isolates can be classified into seven main geographical groups: three African, two Asian and two European [21]. As also shown by us, the three African clades include isolates mainly obtained in western (Africa 1), central (Africa 2) and southern Africa (Africa 3); the two Asian clades encompass isolates from the Middle East and Pakistan (Asia 1) or from China and central Asia (Asia 2). Most of the isolates causing recent outbreaks in eastern Europe are in the Europe 1 clade, whereas Europe 2 has largely divergent strains isolated from ticks in Greece (including the prototype strain AP92) [34] and Turkey [35] that are currently associated with sub-clinical or mild cases. The strong spatial structure of the genetic variability of CCHFV suggested it would be worth studying the migration of the clades phylogeographically in order to reconstruct the possible origin and dispersion pathways of the different viral strains. A study published two years ago [22] used a phylogeographical approach based on maximum parsimony, but new Bayesian methods have recently been developed that allow simultaneous estimates of evolutionary rates and migration routes [31], which are useful for reconstructing the past and recent epidemiological history of highly variable viruses [36-38] 

In order to calculate the tMRCAs for the root and internal nodes of the phylogeographical tree using a relaxed molecular clock model, we estimated a mean evolutionary rate of 2.96x10^-4^ for the viral genomic S segment. This suggests that, like the majority of other RNA viruses, CCHFV is characterised by a rapid evolution, at least at the level of the S fragment.

Our phylodynamic and phylogeographical reconstruction suggests that the known CCHFV clades shared a common ancestor that existed about one thousand years ago, most probably in Africa. A possible origin in Western Africa was suggested by our preliminary analyses [data not shown], but the extensive geographical intermixing of the three African clades and the relatively small number of African isolates available prevented us from reaching any more precise conclusions. 

In our reconstruction, CCHFV left Africa in the second half of the XVII century, reached the Middle East, and then dispersed in two directions in the early XIX century to form the two Asian clades: one spreading in Iran and Pakistan, and the second in China and central Asia (Uzbekistan, Tajikistan). The virus therefore originally spread in an eastward direction to the Middle East and south-east Asia, crossing an area with a constant presence of CCHF susceptible species and the Himalayan mountains as a barrier to natural dispersion. It has recently been speculated that pathogens spread along the Eurasian ruminant route, as in the cases of foot-and-mouth disease [39,40] and Rinderpest [41]. 

Two highly divergent CCHFV strains entered Europe on at least two occasions: the first in the early 1800s (when a less or non-pathogenic virus confined to the animal/vector population reached Greece and Turkey), and the second in the first decades of the XX century, when a more pathogenic strain caused human outbreaks in eastern Europe until recently. In our phylogeographical reconstruction, the most probable location of the MRCA of this European clade 1 was Russia, which suggests that this was the gateway through which genotype 4 CCHFV entered Europe in the early 1900s. This partially conflicts with previous findings [22] suggesting that Turkey was the origin of genotype 4, although our analysis of significant migration rates confirmed the central role of Turkey in spreading the virus throughout Europe. Interestingly, the maximum parsimony analysis showed in- and outflows to and from Turkey and Russia, which suggests a continuous exchange between neighboring areas in the region of the Black Sea (possibly justifying the partial discrepancies between studies), and a complex web of viral introductions/transmissions from Turkey to Kosovo, and from Kosovo to Albania. 

A recent study [42] has suggested the importance of anthropogenic factors in the dispersion of tick-borne encephalitis virus (TBEV), and provided evidence that its temporal and spatial distribution was related to the first land road into Siberia and the trans-Siberian railway. It is interesting to note that the entire area between the Black and Caspian Seas in the time span estimated for the origin of CCHFV (the first decades of the XX century) was the theatre of a number of wars (i.e. the Crimean war, World War II, and the Civil war) and mass deportations. The virus later spread to Turkey, where it probably circulated in the wildlife population for a long time (as previously postulated by [22 ]) as the first human cases of hemorrhagic fever were reported in 2002 [8]. 

In our reconstruction, the virus spread from Turkey to the Balkans, reaching Kosovo in the 1990s and Albania in the last decade. It is possible to hypothesise that the main cause of its dispersion through eastern Europe was the movement of wild and domestic ungulates carrying infected ticks, although outbreaks of CCHFV infection in South Africa have been associated with the passive transportation of infected ticks by birds [9,43], and the same has recently been suggested as introducing the virus to Spain [13]. Although the region between Black and Caspian Seas is one of the major migratory routes for birds crossing the African-Eurasian flyways (http://www.birdlife.org/), movements from East to West are extremely rare [44].

Turkey has one of the largest ruminant populations in Europe and the Middle East, and witnesses the movement of large and small ruminants for breeding, transhumance (within and across its borders), slaughter and import/export. The main direction of the flow is from neighboring countries such as Russia and Iran [45] and the regions of eastern and central Anatolia to the West. Movements take place throughout the year, but especially before and during religious festivals, when there is an increase in the export of small ruminants [46]. This large-scale movement of livestock over a short period of time has been previously associated with CCHF, Rift Valley fever and *peste-des-petits ruminants* [47]. Moreover, cross-border transhumance occurs on the Russian border [39], and could explain the results of the maximum parsimony analysis of in- and outflows between Turkey and Russia. 

The currently used methods of phylogeographical reconstruction are inherently limited by the availability of sample locations and the numbers of isolates at each location. The sensitivity test performed in this study (which suggested that sampling frequencies had little impact on the root location) cannot exclude the influence of unsampled locations. Nevertheless, the analysed data set included all of the sequences with a known sampling location and year that were available in public databases at the time the study began. 

In particular, the scarcity of sequences from Bulgaria prevented us from fully clarifying the country’s role in disseminating the infection. Bulgarian Thrace and Thrace as a whole (a geographical area covering south-eastern Bulgaria, north-eastern Greece and the European part of Turkey) is a high-risk zone for the cross-border spread of animal infectious diseases, as has recently been reported in the case of outbreaks of foot-and-mouth disease in Bulgaria and Greece close to the Turkish border [46]. Moreover, the emergence of human CCHF cases in Thrace over a limited period of time and encompassing three different countries -Turkey [48], Bulgaria and Greece [49,50]- confirm the key role of this area in the spread of infectious diseases.

The findings of this study indicate that continuous surveillance of the CCHF epidemic in Turkey and the entire Thracian area may be very important for monitoring and predicting future CCHF outbreaks in the Balkans.

## Supporting Information

Figure S1 **Evaluation of the impact of sampling heterogeneity on the phylogeographic reconstruction.** The figure shows the root state probability as a function of the location sample size. Randomisation analysis of the tip-localities throughout the MCMC analysis revealed a low level of correlation between the number of taxa per locality and the root-location probability.
(PPTX)

Figure S2 **Likelihood map of the 121 CCHFV S gene sequences.** Each dot represents the likelihoods of the three possible unrooted trees per quartet randomly selected from the data set: the dots near the corners and sides respectively represent tree-like (fully resolved phylogenies in which one tree is clearly better than the others) and network-like phylogenetic signals (three regions in which it is not possible to decide between two topologies). The central area of the map represents a star-like signal (the region in which the star tree is the optimal tree). The numbers indicate the percentage of dots in the centre of the triangle.
(JPG)

Figure S3 **Maximum likelihood tree of the 121 CCHFV S gene sequences.** The numbers on the branches represent bootstrap values (see Materials and Methods for details). The previously described viral genotypes [22] have been highlighted.
(JPG)

Table S1 **Accession numbers and characteristics of the CCHFV sequences used in the study.**
(DOCX)
