# Supplementary figures and images for: Bayesian Phylogeography of Crimean-Congo Hemorrhagic Fever Virus in Europe
Source: PLoS One. 2013 Nov 4;8(11):e79663. doi: 10.1371/journal.pone.0079663 (PMC3817137; doi:10.1371/journal.pone.0079663)

## Slide 1
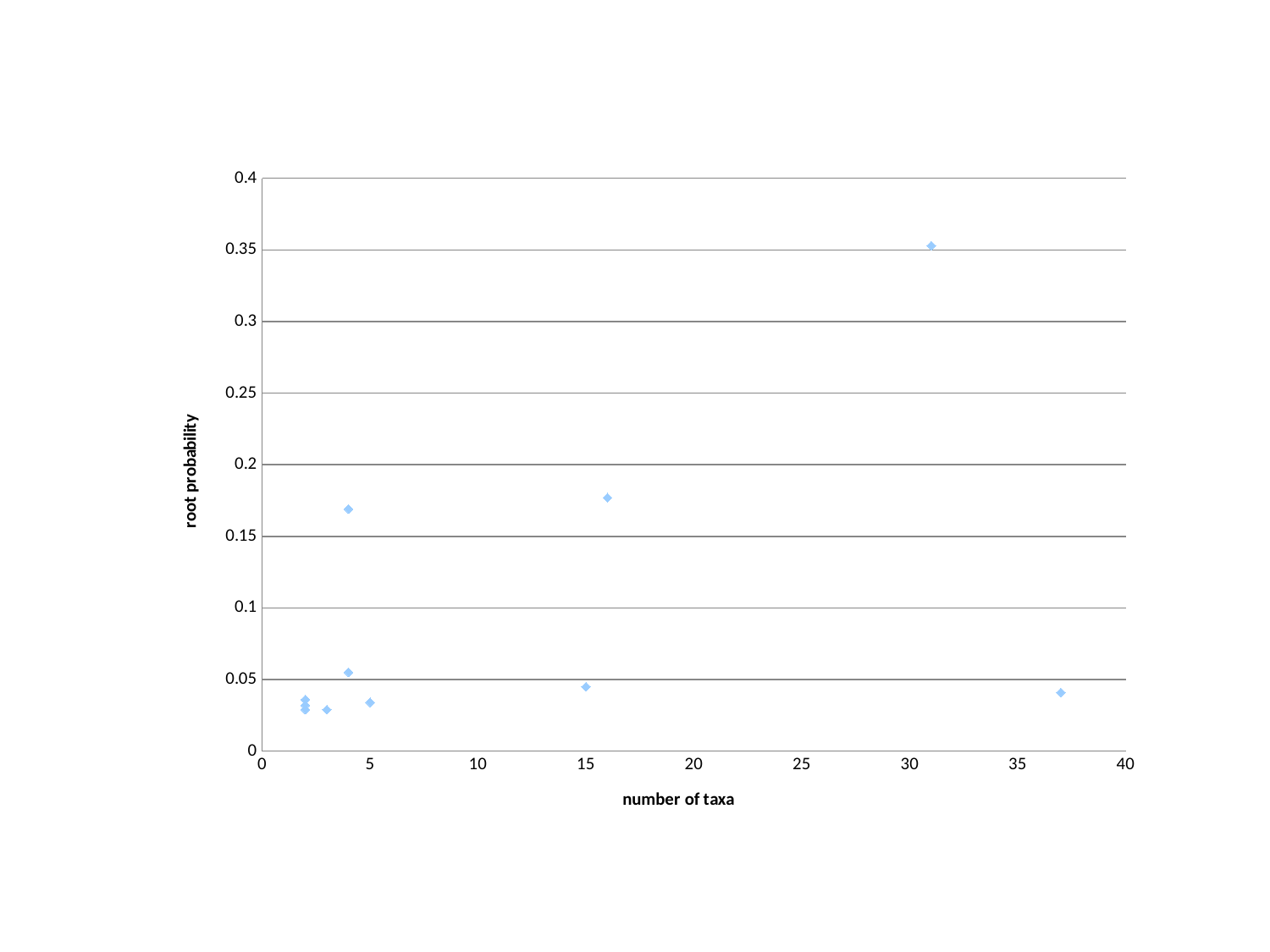

### Chart
| Category | |
|---|---|

Supplement: Figure S1 — Evaluation of the impact of sampling heterogeneity on the phylogeographic reconstruction. The figure shows the root state probability as a function of the location sample size. Randomisation analysis of the tip-localities throughout the MCMC analysis revealed a low level of correlation between the number of taxa per locality and the root-location probability. (PPTX) [file pone.0079663.s001.pptx]

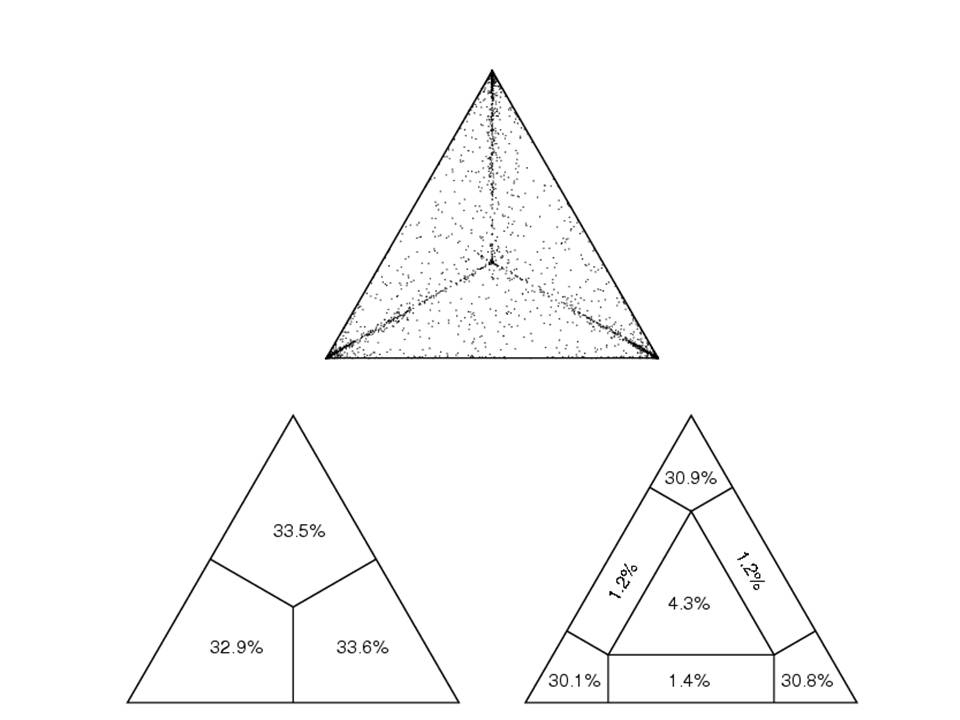

Supplement: Figure S2 — Likelihood map of the 121 CCHFV S gene sequences. Each dot represents the likelihoods of the three possible unrooted trees per quartet randomly selected from the data set: the dots near the corners and sides respectively represent tree-like (fully resolved phylogenies in which one tree is clearly better than the others) and network-like phylogenetic signals (three regions in which it is not possible to decide between two topologies). The central area of the map represents a star-like signal (the region in which the star tree is the optimal tree). The numbers indicate the percentage of dots in the centre of the triangle. (JPG) [file pone.0079663.s002.jpg]

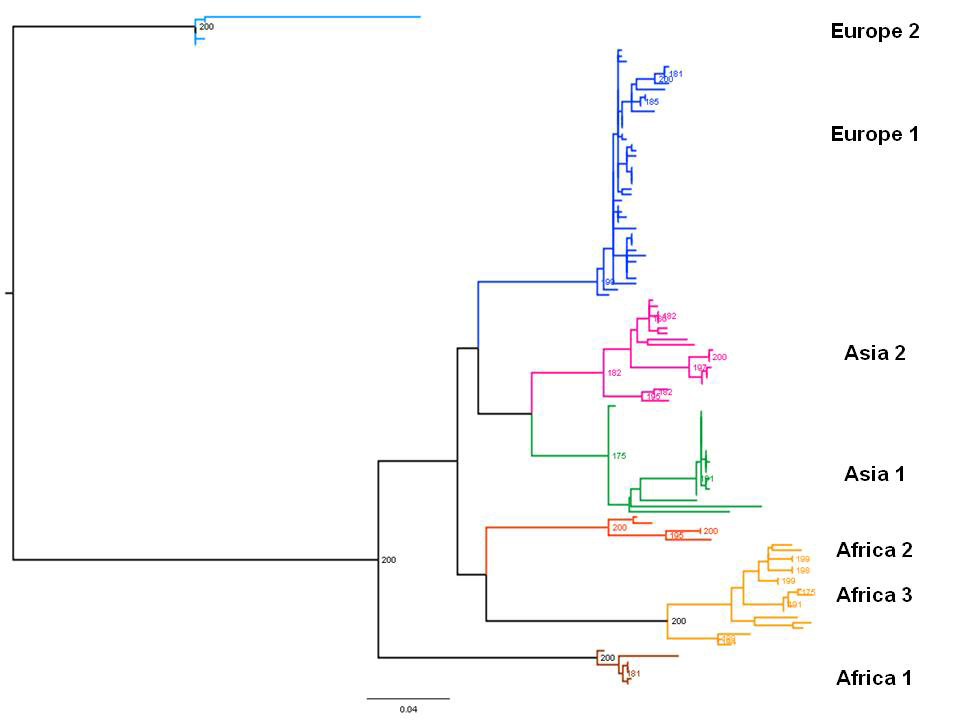

Supplement: Figure S3 — Maximum likelihood tree of the 121 CCHFV S gene sequences. The numbers on the branches represent bootstrap values (see Materials and Methods for details). The previously described viral genotypes [22] have been highlighted. (JPG) [file pone.0079663.s003.jpg]
