# Supplementary material for: Bayesian Phylogeography of Crimean-Congo Hemorrhagic Fever Virus in Europe
Source: PLoS One. 2013 Nov 4;8(11):e79663. doi: 10.1371/journal.pone.0079663 (PMC3817137; doi:10.1371/journal.pone.0079663)
Supplement: Table S1 — Accession numbers and characteristics of the CCHFV sequences used in the study. (DOCX) [file pone.0079663.s004.docx]

**Table S1- Accession numbers and characteristics of the CCHFV sequences used in the study.**

| **Sample** | **Accession number** | **Isolate** | **Sampling Location** | **Host** | **Sampling Year** |
| --- | --- | --- | --- | --- | --- |
| AP92@75 | U04958 | AP92 | Greece | Rhipicephalus bursa | 1975 |
| SE1@93 | U15091 | ArD 97268 | Senegal | H. truncatum | 1993 |
| SE2@93 | U15090 | ArD 97264 | Senegal | H. marginatum rufipes | 1993 |
| SE3@72 | DQ211640 | ArD15786 | Senegal | goat | 1972 |
| SE4@69 | DQ211639 | ArD8194 | Senegal | H. truncatum | 1969 |
| SE5@69 | U88411 | DAK 8194 | Senegal |  | 1969 |
| SE6@69 | U15021 | ArD 8194 | Senegal |  | 1969 |
| UGA1@56 | U88416 | UGANDA 3010 | Uganda | human | 1956 |
| UGA2@81 | DQ076415 | SPU128/81/7 | Uganda | Hyalomma (tick) | 1981 |
| UGA3@58 | DQ076413 | Semunya | Uganda |  | 1958 |
| CAR1@73 | U15092 | ArB 604 | CAR | H.nitidum(tick) | 1973 |
| NIG1@66 | U88410 | IbAr10200 | Nigeria | H. excavatum (tick) | 1966 |
| MAD1@85 | U15024 | ArMg 951 | Madagascar | Boophilus microplus (tick) | 1985 |
| SA1@86 | U84638 | SPU 422/86 | South Africa |  | 1986 |
| SA2@87 | DQ211647 | SPU103/87 | South Africa | human | 1987 |
| SA3@85 | DQ211646 | SPU97/85 | South Africa | human | 1985 |
| SA4@89 | FJ435421 | SPU 337/89 | South Africa | human | 1989 |
| SA5@88 | FJ435422 | SPU 45/88 | South Africa | human | 1988 |
| SA6@88 | FJ435423 | SPU 71/88 | South Africa | human | 1988 |
| SA7@81 | DQ076416 | SPU4/81 | South Africa | human | 1981 |
| SA8@85 | U84636 | SPU 247/85 | South Africa |  | 1985 |
| SA9@86 | U84635 | SPU 582/86 | South Africa |  | 1986 |
| SA10@88 | U84637 | 45/88 | South Africa |  | 1988 |
| SA11@85 | DQ211648 | SPU415/85 | South Africa | human | 1985 |
| MAU1@84 | U15089 | ArD 39554 | Mauritania | H. marginatum rufipes | 1984 |
| MAU2@84 | DQ211641 | ArD39554 | Mauritania | H. marginatum rufipes | 1984 |
| MAU3@88 | U15023 | HD 49199 | Mauritania | human | 1988 |
| CO1@56 | DQ211650 | UG3010 | Congo | human | 1956 |
| SUD1@08 | GQ862371 | Sudan Al-Fulah 3-2008 | Sudan | human | 2008 |
| SUD2@08 | GQ862372 | Sudan Al-Fulah 4-2008 | Sudan | human | 2008 |
| SUD3@09 | HQ378179 | Sudan AB1 | Sudan | human | 2009 |
| BF1@83 | U15093 | HD 38562 | Burkina Faso | human | 1983 |
| OMN1@97 | DQ211645 | Oman strain | Oman |  | 1997 |
| AL5@04 | KC846094 | 178/04 | Albania | human | 2004 |
| AL6@03 | KC846093 | Albania 23/03 | Albania | human | 2003 |
| KO1@00 | AF404507 | Kosovo strain | Kosovo | human | 2000 |
| KO2@01 | AF428144 | 9553/01 | Kosovo | human | 2001 |
| KO4@01 | DQ133507 | Kosovo Hoti | Kosovo | human | 2001 |
| BU9@08 | FJ472634 | Bul2008 | Bulgaria | human | 2008 |
| BU10@78 | AY277676 | Bul/hU517 | Bulgaria | human | 1978 |
| T1@06 | DQ983741 | CTF-Hu7/06 | Turkey | human | 2006 |
| T2@06 | GQ337053 | Turkey-Kelkit06 | Turkey | human | 2005 |
| T3@08 | FJ392604 | KMAG-Hu-08-01 | Turkey | human | 2008 |
| T4@07 | EU727456 | GOU-OT07 | Turkey | human | 2007 |
| T5@03 | DQ211649 | Turkey200310849 | Turkey | human | 2003 |
| T6@05 | EF432649 | Kelkit S/Turkey-hu11/2005 | Turkey | human | 2005 |
| T7@04 | EF432640 | Kelkit S/Turkey-hu1/2004 | Turkey | human | 2004 |
| T8@08 | FJ601895 | 1503 | Turkey | human | 2008 |
| T9@08 | FJ601863 | BOLU173-2006-201 | Turkey | human | 2008 |
| Tke10@04 | EF432639 | hu2/2004 | Turkey | human | 2004 |
| Tke11@04 | EF432653 | hu15/2004 | Turkey | human | 2004 |
| Tke12@05 | EF432650 | hu12/2005 | Turkey | human | 2005 |
| Tke13@05 | EF432647 | hu9/2005 | Turkey | human | 2005 |
| Tky19@09 | HQ188915 | Kayseri 3159-2009 | Turkey | human | 2009 |
| Tay20@10 | HQ188918 | Aydin 193-2010 | Turkey | human | 2010 |
| Tay21@09 | HQ173901 | Aydin 919-2009 | Turkey | human | 2009 |
| Tyo22@10 | HQ188920 | Yozgat 714-2010 | Turkey | human | 2010 |
| Tto24@06 | GU324990 | k16-2-kk536 | Turkey | tick | 2006 |
| Tto25@06 | GU324991 | k36-kk536 | Turkey | tick | 2006 |
| Tto26@06 | GU324992 | k52-kk536R | Turkey | tick | 2006 |
| Tto27@06 | GU324993 | k53-kk536 | Turkey | tick | 2006 |
| Tto28@09 | HQ173902 | Tokat 1728-2009 | Turkey | human | 2009 |
| Tar29@10 | HQ675002 | Artvin 1913-2010 | Turkey | human | 2010 |
| Tba30@09 | HQ821873 | BAYBURT 2946-2009 | Turkey | human | 2009 |
| Tca31@09 | HQ675006 | Cankiri 1079-2009 | Turkey | human | 2009 |
| Tco32@09 | HQ675010 | Corum 4319-2009 | Turkey | human | 2009 |
| Ter33@10 | HQ664913 | Erzurum 1912-2010 | Turkey | human | 2010 |
| Tez34@10 | HQ675008 | Erzincan 1910-2010 | Turkey | human | 2010 |
| Tka35@10 | HQ675007 | Karabuk 590-2010 | Turkey | human | 2010 |
| Tks36@09 | HQ173895 - | Kastamonu 1420-2009 | Turkey | human | 2009 |
| Tsa37@09 | HQ675003 | Samsun 1030-2009 | Turkey | human | 2009 |
| Tsi38@09 | HQ173893 | Sivas 405-2009 | Turkey | human | 2009 |
| Tel39@06 | DQ983785 | ZAM57/06 | Turkey | H.marginatum | 2006 |
| I1@78 | U15022 | ArTeh 193-3 | Iran |  | 1978 |
| Isi2@02 | AY366373 | 766/02 | Iran | human | 2002 |
| Isi3@02 | AY366374 | 756/02 | Iran | human | 2002 |
| Isi4@02 | AY366376 | 714/02 | Iran | human | 2002 |
| Isi5@02 | AY366377 | 782/02 | Iran | human | 2002 |
| Isi6@02 | AY366378 | 786/02 | Iran | human | 2002 |
| Isi7@02 | AY366379 - | 787/02 | Iran | human | 2002 |
| Iha8@07 | GU456723 | CT9 | Iran | Rhipicephalus sanguineus | 2007 |
| Iha9@07 | GU456724 | CT10 | Iran | H. detritum (tick) | 2007 |
| Iha10@07 | GU456725 | CT12 | Iran | H. detritum (tick) | 2007 |
| Iha11@07 | GU456726 | CT13 | Iran | Argas reflexus (tick) | 2007 |
| Iha12@07 | GU456727 | CT14 | Iran | H. anatolicum (tick) | 2007 |
| Iha13@07 | GU456728 | CT15 | Iran | H. detritum (tick) | 2007 |
| Iqo14@02 | AY366375 | 729/02 | Iran | human | 2002 |
| IRQ1@79 | AJ538196 | Baghdad 12 | Iraq | human | 1979 |
| PAK1@65 | U88414 | JD 206 | Pakistan | H. anatolicum | 1965 |
| PAK2@76 | AF527810 | Matin | Pakistan |  | 1976 |
| PAK4@00 | AJ538198 | SR3 | Pakistan | human | 2000 |
| AFG1@09 | HM452305 | Afg09 | Afghanistan | human | 2009 |
| UZB1@85 | AF481799 | Uzbek/TI10145 | Uzbekistan | H. asiaticum | 1985 |
| UZB2@67 | AY223475 | Hodzha | Uzbekistan | human | 1967 |
| TAJ1@90 | AY049083 | TAJ/HU8966 | Tajikistan | human | 1990 |
| TAJ3@91 | AY297691 | TAJ/HU8978 | Tajikistan | human | 1991 |
| R1@67 | DQ211644 | Kashmanov | Russia | human | 1967 |
| Rst2@00 | AF481802 | STV/HU29223 Russia | Stavropol | human | 2000 |
| Ras6@67 | DQ211643 | Drosdov | Astrakhan | human | 1967 |
| Rro15@00 | AY277672 | ROS/TI28044 | Rostov | H. marginatum | 2000 |
| Rro16@02 | DQ206447 | ROS/HUVLV-100 Russia | Rostov | human | 2002 |
| CH8@04 | GU477494 | 79121M18 | China | tick | 2004 |
| CH9@04 | FJ562093 | YL04057 | China | tick | 2004 |
| CH10@05 | DQ227496 | CLT/TI05146 | China | tick | 2005 |
| CH11@05 | DQ227495 | CYT/TI05099 | China | tick | 2005 |
| CH12@05 | DQ217602 | CYL/TI05035 | China |  | 2005 |
| CH13@68 | M86625 | C68031 | China |  | 1968 |
| CH14@70 | AF415236 | c7001 | China |  | 1970 |
| CH15@79 | AF358784 | c79121 | China |  | 1979 |
| CH16@66 | AJ010648 | c66019 | China | human | 1966 |
| CH17@88 | AY029157 | c88166 | China |  | 1988 |
| CH18@84 | AJ010649 | c8402 | China | H. asiaticum | 1984 |
| CH19@68 | DQ211642 | C-68031 | China | sheep | 1968 |
| CH20@75 | AF362080 | c75024 | China |  | 1975 |
| CH21@78 | AF354296 | c7803 | China |  | 1978 |
| CH22@68 | U88413 | HY 13 | China | H. asiaticum | 1968 |
| T40@08 | FJ392604 | KMAG-Hu-08-01 | Turkey | human | 2008 |
| T41@08 | FJ392601 | Tr-T-03 | Turkey | Rhipicephalus bursa | 2008 |
| T42@08 | FJ392603 | Tr-T-11 | Turkey | Rhipicephalus bursa | 2008 |
| Tis23@07 | EU057975 | KMAG-Hu-07-01 | Turkey | human | 2008 |
| G1@08 | EU871766 | 66/08-Rodopi | Greece | human | 2007 |
|  |  |  |  |  | 2008 |
